# Risk factors for postpartum stress urinary incontinence: a prospective study

**DOI:** 10.1186/s12894-024-01430-x

**Published:** 2024-02-16

**Authors:** Wei Liu, Linxue Qian

**Affiliations:** Department of Ultrasound, Beijing Friendship Hospital, Capital Medical University, Beijing, China

**Keywords:** Urinary incontinence, Stress, Postpartum period, Pelvic floor, Risk factors

## Abstract

**Purpose:**

Postpartum stress urinary incontinence (SUI) is a common occurrence in women, and it has a profound effect on women’s health and quality of life. This study aimed to investigate the risk factors for postpartum SUI and the relative importance of each factor, including pelvic floor ultrasound measurement data and clinical data.

**Method:**

Pregnant women who delivered in our hospital from March 2021 to January 2022 were selected as the study population. The clinical and anatomical Data from women with SUI and those without SUI were collected and analyzed. The clinical and anatomical risk factors associated with postpartum SUI were identified using univariate and multivariate analyses.

**Results:**

A total of 255 participants were recruited. Logistic regression analysis indicated that age (OR:1.215, 95% CI:1.097–1.346, *P* < 0.001), vaginal delivery (OR:3.05, 95% CI:1.328–7.016, *P* < 0.009), parity (OR:3.059, 95% CI:1.506–6.216, *P* < 0.002), bladder neck descent (OR:4.159, 95% CI: 2.010–8.605, *P* < 0.001), the angle of the internal urethral orifice funnel (OR:1.133, 95% CI:1.091–1.176, *P* < 0.001) were important independent risk factors for postpartum SUI (all *P* < 0.05). The AUC was 0.883 (95% CI: 0.839–0.926) in the model.

**Conclusions:**

Age, vaginal delivery, parity, bladder neck descent and the angle of the internal urethral orifice funnel are independent risk factors for postpartum SUI. To prevent the occurrence of postpartum SUI, high-risk factors of postpartum SUI should be identified as early as possible during pregnancy and after delivery, and postpartum pelvic floor rehabilitation training should be promoted.

## Introduction

Postpartum Stress incontinence (SUI) is a common condition in women. Postpartum SUI can have a serious effect on women’s health and quality of life. Pregnancy and childbirth can affect the structure of the pelvic floor and increase the risk of pelvic floor disorders. Postpartum SUI is a common pelvic floor dysfunction. The current prevailing theory for postpartum SUI is that the destruction of the supporting connective tissue of the bladder and urethra, as well as the weakening of the muscular structure of the pelvic floor, bladder neck and urethral sphincter, lead to reduced urethral closure pressure. This situation functionally leads to postpartum SUI [1]. The International Association of Urogynecology and the International Incontinence Society (ICS) define stress incontinence as involuntary incontinence caused during exertion, such as physical exertion, sneezing or coughing [2]. The incidence of postpartum SUI is as high as 18% [3]. Identifying the risk factors associated with postpartum SUI, which may play a major role in preventing the occurrence of urinary incontinence and provide a basis for preventing and intervening in postpartum SUI, is important.

## Material and methods

### Patients and data collection

This study was approved by the Ethics Committee of Beijing Friendship Hospital affiliated with Capital Medical University (YYYXYJ-2021–289). The study was performed in accordance with the Declaration of Helsinki. We selected pregnant women who delivered in our hospital from March 2021 to January 2022 as the study population.

The inclusion criteria were as follows: (1) women ≥ 18 years of age, (2) full-term singleton delivery, (3) no history of pelvic surgery or urinary system diseases, and (4) clear awareness and willingness to participate in the study. The exclusion criteria for this study were as follows: (1) diabetes, hypertension; (2) severe cardiovascular and pulmonary diseases; (3) a history of pelvic and vaginal surgeries; and (4) prepregnancy urinary incontinence.

### SUI diagnostic criteria

SUI was diagnosed if the participants responded “yes” to the question “Did you leak urine with activities, such as coughing, sneezing, or running?” [4, 5].

### Perineal ultrasound evaluation and image analysis

All images were obtained using the GE voluson E10 and Mindray Resona R7, and the participants underwent a pelvic floor ultrasound examination 6–12 weeks after delivery. The pelvic floor image was placed in the center of the screen to obtain the midsagittal section image, and obtain and analyze images in resting, contracting and maximum Valsalva states. The collected ultrasound data included bladder neck mobility, the angle of the internal urethral orifice funnel, the urethral rotation angle, the retrovesical angle, the levator hiatal area, and thickness of the left and right puborectalis muscles. During the collection process, we avoided excessive pressure on the probe that might cause co-activation of the levator ani muscle. The Valsalva action state lasted for at least 6 s, and we collected data at least three times to obtain satisfactory maximum Valsalva state volume data. All data were integrated for further analysis.

### Statistical analysis

Statistical analysis was performed using SPSS software version 23.0 (IBM, Armonk, NY, USA), R software 3.6.0 (The R Foundation for Statistical Computing). The association between candidate predictive variables and SUI was evaluated using a univariate analysis. In quantitative data, use median and IQRs to represent. The χ^2^ test was used to compare the classified data. A multivariate logistic regression analysis was used to determine the risk factors for SUI. The odds ratio (ORs) and their associated 95% confidence intervals (CIs) were calculated. A receiver operating characteristic (ROC) curve analysis was used to determine the optimal threshold from the area under the curve. The sensitivity, specificity, accuracy, positive predictive value and negative predictive value of each index in the diagnosis of postpartum SUI were calculated.

## Results

We recruited 255 postpartum women to participate in this study. Among them, there were 105 women in the case group (with SUI) and 150 women in the control group (without SUI).

### Univariate and multivariate logistic regression analyses

**Fig. 1:**
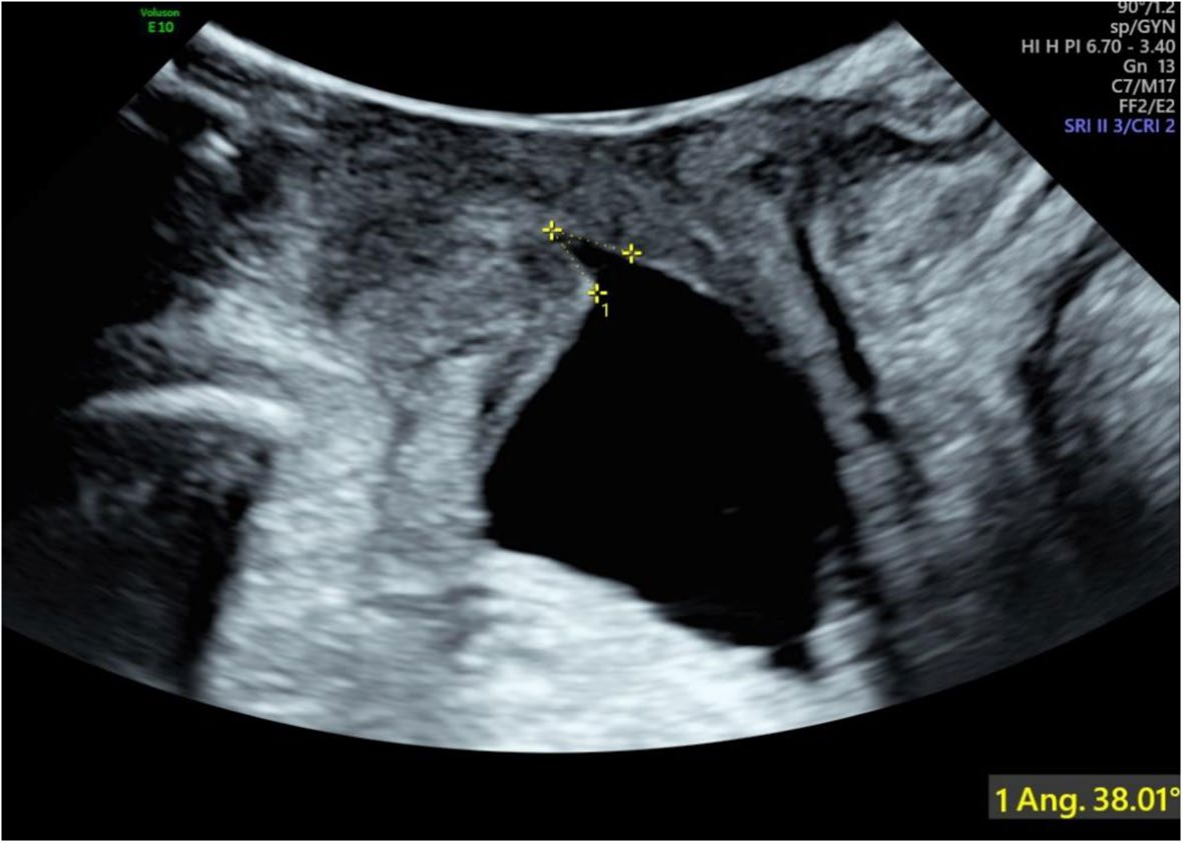
In the maximum Valsalva maneuver, a funnel is formed at the urethral opening at an angle of 38.01°

**Fig. 2:**
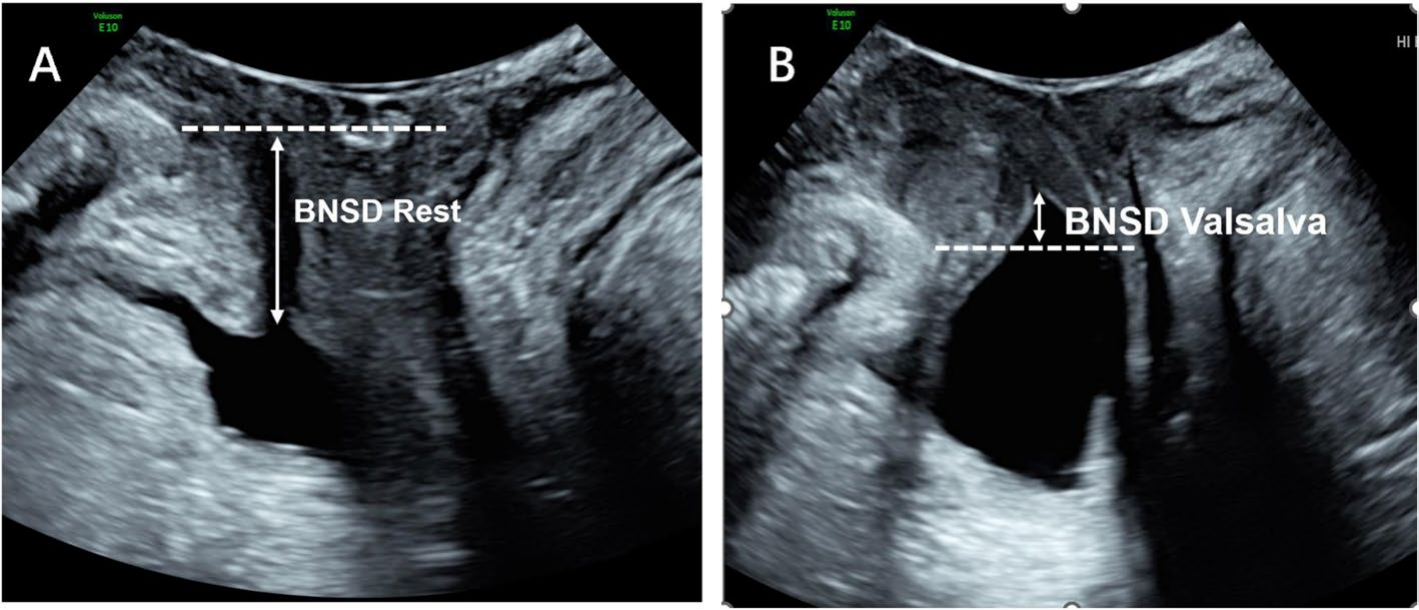
**A** At rest, the distance between the bladder neck and the pubic symphysis is 1.6 cm; **B** The distance between the bladder neck and the pubic symphysis in Valsalva state is -0.4 cm; The mobility of the bladder neck is 1.6 cm—(-0.4 cm) = 2.0 cm

**Fig. 3:**
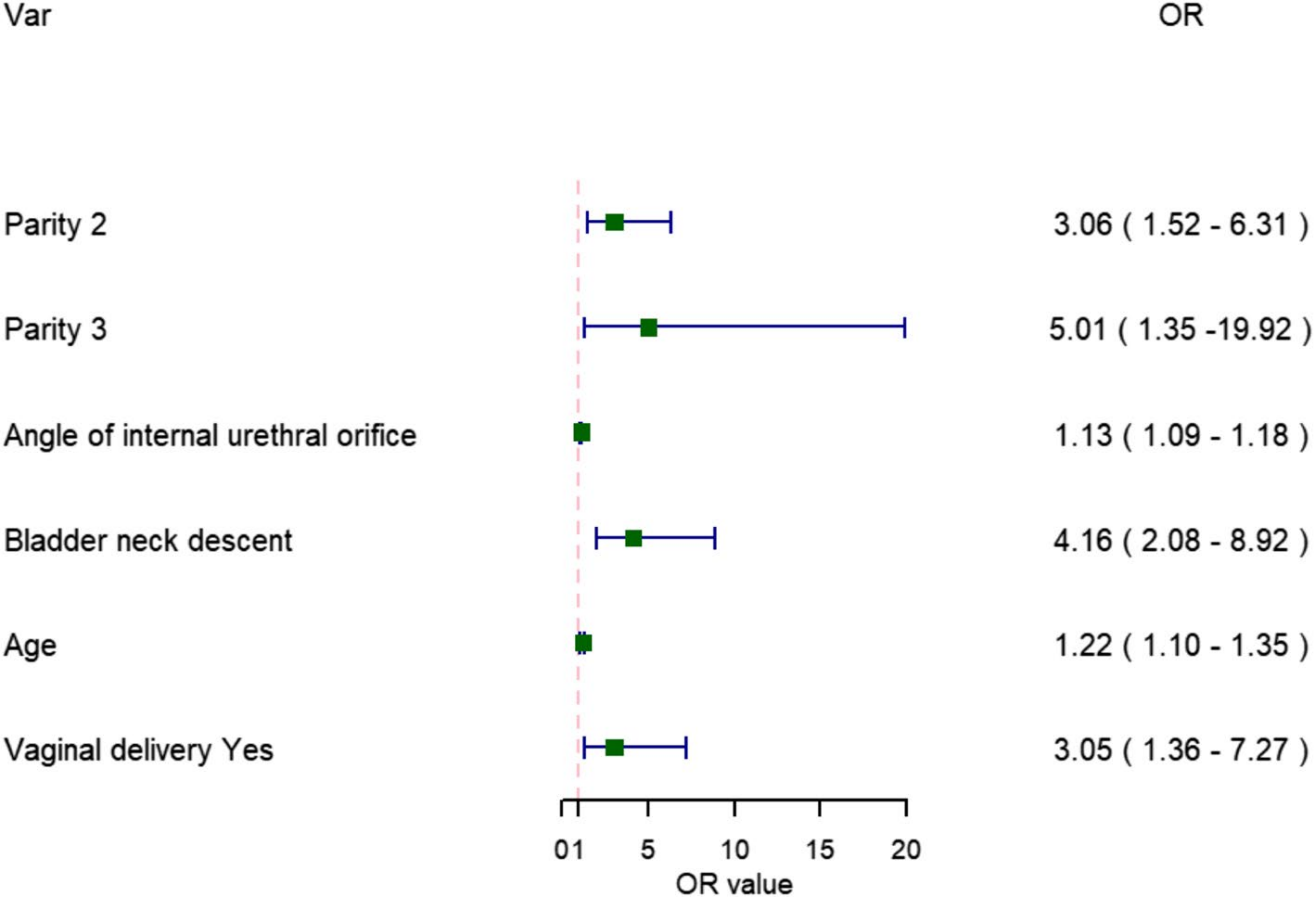
Forest plot of independent predictors of postpartum SUI using multiple regression models

There were significant differences in clinical data (age, delivery mode and parity) between the case group and control group (all *P* < 0.05). There were significant differences in anatomical measurement parameters of the pelvic floor (bladder neck movement and the angle of the internal urethral orifice funnel) between the groups (both *P* < 0.05) (Figs. 1, 2). The results showed that the age, vaginal delivery, bladder neck movement and the angle of the internal urethral orifice funnel in the case group (with SUI) and control group (without SUI) were 31.0 (29.0–35.0) vs 30.0 (27.8–32.0), 92 (87.62%) vs 85 (56.67%), 1.5 (1.3–2.2) vs 1.3 (1.2–1.5), 24.0 (16.0–34.5) vs 12.5 (7.0–18.0), respectively. Logistic regression analysis indicated that age (OR:1.215, 95% CI:1.097–1.346, *P* < 0.001), vaginal delivery (OR:3.05, 95% CI:1.328–7.016,P = 0.009), parity (OR:3.059, 95% CI:1.506–6.216, *P* = 0.002), bladder neck descent (OR:4.159, 95% CI: 2.010–8.605, *P* < 0.001), the angle of the internal urethral orifice funnel (OR:1.133, 95% CI:1.091–1.176,P < 0.001) were important independent risk factors for postpartum SUI. (In Table 1, Fig. 3) The AUC was 0.883 (95% CI: 0.839–0.926) in the model. +

**Table 1:** Univariate Analysis and multivariate logistic regression analysis of clinical and ultrasonic characteristics

Variable	Univariate Analysis				Multivariate Analysis	
	SUI (n = 105)	Non-SUI (n = 150)	Statistic	*P*	OR (95%CI)	*P*
Age	31.0 (29.0 ~ 35.0)	30.0 (27.8 ~ 32.0)	-4.671	< 0.001	1.215 (1.097–1.346)	< 0.001
Parity						
1	35 (33.33%)	95 (63.33%)	22.56	< 0.001	Ref	–
2	60 (57.14%)	49 (32.67%)			3.059 (1.506–6.216)	0.002
3	10 (9.52%)	6 (4%)			5.014 (1.322–19.016)	0.018
Vaginal delivery						
Yes	92 (87.62%)	85 (56.67%)	27.87	< 0.001	3.053 (1.328–7.016)	0.009
No	13 (12.38%)	65 (43.33%)				
Angle of internal urethral orifice funnel	24.0 (16.0 ~ 34.5)	12.5 (7.0 ~ 18.0)	-8.117	< 0.001	1.133 (1.091–1.176)	< 0.001
Bladder neck descent	1.5 (1.3 ~ 2.2)	1.3 (1.2 ~ 1.5)	-3.480	< 0.001	4.159 (2.010–8.605)	< 0.001
Urethral rotation angle	20.0 (15.0 ~ 26.0)	19.0 (15.0 ~ 25.0)	-0.702	0.483		
Retrovesical angle	150.0 (145.0 ~ 157.0)	150.0 (144.8 ~ 156.0)	-0.898	0.369		
Levator hiatal area	18.5 (15.5 ~ 19.9)	16.5 (15.2 ~ 19.0)	-1.717	0.086		
Thickness of left puborectalis muscle	1.00 (0.90 ~ 1.05)	0.98 (0.82 ~ 1.08)	-0.716	0.474		
Thickness of right puborectalis muscle	0.98 (0.90 ~ 1.11))	1.00 (0.90 ~ 1.10)	-1.658	0.097

### ROC curve analysis

**Fig. 4:**
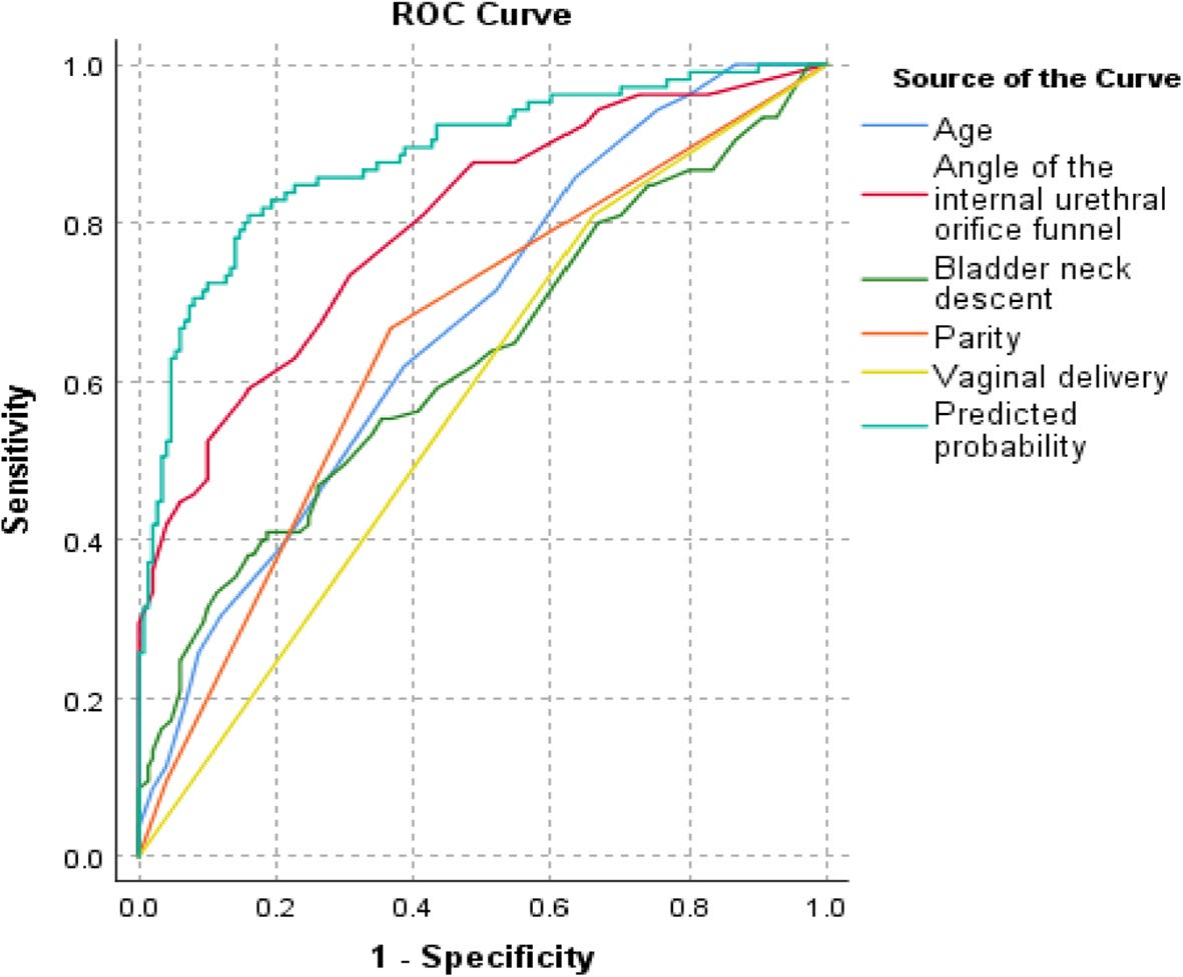
Receiver operating characteristic (ROC) curves. The predictors of SUI were age, parity, vaginal delivery, bladder neck descent, and angle of internal urethral orifice funnel. The areas under the curve (AUCs) of these factors ranged from 0.575 to 0.798

We analyzed the ROC curves of each independent risk factor and evaluated their ability to diagnose postpartum SUI. The area under the curve, sensitivity, specificity, accuracy, positive predictive value and negative predictive value of each independent risk factor are shown in Table 2. The areas under the curve of each variable (parity, age, vaginal delivery, bladder neck mobility and funnel angle) were 0.654, 0.671, 0.575, 0.628 and 0.798, respectively. According to the ROC, age > 31 years old, bladder neck mobility > 1.88 cm, and a funnel angle > 19.5° were the optimal cutoff values for postpartum SUI. (In Table 2, Fig. 4).

**Table 2:** ROC analyses of independent factors for predicting SUI

Parameter	Area	95%CI	Sensitivity	Specifity	Accuracy	PPV	NPV
Predictive equation	0.883	0.839–0.926	0.810	0.840	0.827	0.780	0.863
Age	0.671	0.605–0.737	0.619	0.613	0.616	0.528	0.697
Vaginal delivery	0.575	0.504–0.645	0.810	0.340	0.533	0.462	0.718
Parity	0.654	0.586–0.72	0.667	0.633	0.647	0.56	0.731
Angle of internal urethral orifice funnel	0.798	0.742–0.854	0.590	0.840	0.737	0.721	0.746
Bladder neck descent	0.628	0.557–0.699	0.410	0.813	0.645	0.606	0.663

## Discussion

The structure of the female pelvic floor is composed of pelvic floor muscles, fascia and ligaments, which support the pelvic organs and maintain physiological functions, such as urination and defecation [6]. Pregnancy and childbirth are closely related to the occurrence and development of postpartum SUI [7]. Pregnancy and childbirth can cause damage to the structure and function of the pelvic floor, and can cause pelvic organ prolapse and postpartum SUI [8]. At present, the risk factors for postpartum SUI are unclear. In clinical practice, the assessment of postpartum SUI in women is based on the clinical experience of doctors, but there is a lack of objective evidence. Therefore, this study aimed to evaluate the relationship between clinical factors and pelvic floor ultrasound measurement parameters and postpartum SUI. Our study showed that age, vaginal delivery, parity, bladder neck mobility and the funnel angle were independent risk factors for postpartum SUI. Patients older than 31 years, bladder neck movement > 1.88 cm, a funnel angle > 19.5°, vaginal delivery and parity greater than one require early intervention and prevention to reduce the occurrence of postpartum SUI.

The process of pregnancy and childbirth leads to pelvic floor muscle relaxation and reduced contractile function, which increases the risk of postpartum SUI. In the multiple logistic regression analysis, age was identified as an independent risk factor for postpartum SUI. Our results indicate that older mothers are more prone to postpartum SUI. Therefore, attention should be paid to the possibility of postpartum SUI in older mothers. Postpartum SUI is more pronounced in women with multiple pregnancies than in primiparous women. In this study, the risk of postpartum SUI increased with the number of cesarean or vaginal deliveries. Women with multiple pregnancies have more pronounced urinary incontinence symptoms and pelvic floor structural changes than primiparous women. When a woman becomes pregnant again, the pelvic floor structure is damaged again, and the supporting force of tissues around the urethra is further weakened, thus increasing the occurrence of postpartum SUI.

Vaginal delivery and parity are also important predictive factors for postpartum SUI. Women with a history of vaginal delivery have a higher risk of developing postpartum SUI than those with cesarean section. Some scholars believe that a history of vaginal delivery is an independent risk for postpartum SUI. The process of vaginal delivery, especially with instrument-assisted delivery, can cause damage to the pelvic floor. Delivery has a considerable effect on the anatomical structure and function of the pelvic floor. During childbirth, the pelvic floor is compressed by the fetal head, causing pelvic floor stretching and elongation. When this exceeds the limit of connective tissue extension, it can cause damage to the connective tissue. Childbirth can cause damage to muscles and connective tissue, as well as damage to nerves [9, 10]. In this study, the OR value for vaginal delivery compared with cesarean delivery was 3.053 (95% CI, 1.328–7.016). Cesarean section reduces the damage to pelvic floor muscle tissue and nerves caused by vaginal delivery. Multiple studies have shown that the incidence of SUI in women who deliver vaginally is higher than that in those with cesarean section in the initial postpartum period [11, 12]. Therefore, in women with a history of vaginal delivery, more attention should be paid to screening and prevention.

Pelvic floor ultrasound can clearly show the changes in urethral support structure and bladder neck anatomy in patients with postpartum SUI. This study showed the morphological changes in the bladder, urethra and surrounding tissues through pelvic floor ultrasound images. We also quantified the changes in pelvic floor structure in patients with postpartum SUI by measuring indicators, such as bladder neck mobility and the bladder neck funnel angle. This study suggests that bladder neck mobility and the bladder neck funnel angle are independent risk factors for postpartum SUI. The mobility of the bladder neck is a reliable measurement method for evaluating urethral activity [13]. Previous studies have shown a significant correlation between bladder neck mobility and postpartum SUI, and it increases with the severity of postpartum SUI [4, 14]. In patients with postpartum SUI, the bladder neck or periurethral support ability was weakened, and the internal urethral orifice opened into a funnel shape when abdominal pressure was increased. The angle of the funnel of the internal urethral orifice in the SUI group was significantly different from that in the non-SUI group. The incidence of postpartum SUI increased with an increase in the funnel angle. The formation of the urethral infundibulum is the main ultrasound manifestation of postpartum SUI, and this has high application value in the clinical prediction of postpartum SUI.

The study has some limitations. (1) This study is a single center study with a small sample size and short follow-up time. (2) The parturients involved in this study did not undergo urodynamic examination.

In summary, age, vaginal delivery, parity, bladder neck mobility and the bladder neck funnel angle are independent risk factors for postpartum SUI. To prevent the occurrence of postpartum SUI, high-risk factors of postpartum SUI should be identified as early as possible during pregnancy and after delivery, and postpartum pelvic floor rehabilitation training should be promoted.

## Data Availability

The datasets generated and/or analyzed during the current study are not publicly available due to medical confidentiality regarding patients’ data but are available from the corresponding author on reasonable request.

## Abbreviations

SUI: Stress urinary incontinence
ICS: International Incontinence Society
SD: Standard deviation
OR: Odds ratio
CI: Confidence intervals
ROC: Receiver operating characteristic
